# Acceleration of radiative recombination for efficient perovskite LEDs

**DOI:** 10.1038/s41586-024-07460-7

**Published:** 2024-05-29

**Authors:** Mengmeng Li, Yingguo Yang, Zhiyuan Kuang, Chenjie Hao, Saixue Wang, Feiyue Lu, Zhongran Liu, Jinglong Liu, Lingjiao Zeng, Yuxiao Cai, Yulin Mao, Jingshu Guo, He Tian, Guichuan Xing, Yu Cao, Chao Ma, Nana Wang, Qiming Peng, Lin Zhu, Wei Huang, Jianpu Wang

**Affiliations:** 1 Key Laboratory of Flexible Electronics (KLOFE), Institute of Advanced Materials (IAM) & School of Flexible Electronics (Future Technologies), Nanjing Tech University, Nanjing, China; 2 Strait Institute of Flexible Electronics (SIFE, Future Technologies), Fujian Normal University, Fuzhou, China; 3 School of Microelectronics, Fudan University, Shanghai, China; 4 Center of Electron Microscopy, State Key Laboratory of Silicon Materials, School of Materials Science and Engineering, Zhejiang University, Hangzhou, China; 5 Institute of Applied Physics and Materials Engineering, University of Macau, Macau, China; 6 State Key Laboratory of Extreme Photonics and Instrumentation, College of Optical Science and Engineering, International Research Center for Advanced Photonics, Zhejiang University, Hangzhou, China; 7 Strait Laboratory of Flexible Electronics (SLoFE), Fuzhou, China; 8 Institute of Flexible Electronics (IFE), Northwestern Polytechnical University (NPU), Xi’an, China; 9 MIIT Key Laboratory of Flexible Electronics (KLoFE), Northwestern Polytechnical University (NPU), Xi’an, China; 10 School of Flexible Electronics (SoFE), Sun Yat-sen University, Shenzhen, China; 11 School of Materials Science and Engineering, Changzhou University, Changzhou, China; 12 School of Microelectronics and Control Engineering, Changzhou University, Changzhou, China

**Keywords:** Lasers, LEDs and light sources, Materials for devices

## Abstract

The increasing demands for more efficient and brighter thin-film light-emitting diodes (LEDs) in flat-panel display and solid-state lighting applications have promoted research into three-dimensional (3D) perovskites. These materials exhibit high charge mobilities and low quantum efficiency droop^1–6^, making them promising candidates for achieving efficient LEDs with enhanced brightness. To improve the efficiency of LEDs, it is crucial to minimize nonradiative recombination while promoting radiative recombination. Various passivation strategies have been used to reduce defect densities in 3D perovskite films, approaching levels close to those of single crystals^3^. However, the slow radiative (bimolecular) recombination has limited the photoluminescence quantum efficiencies (PLQEs) of 3D perovskites to less than 80% (refs. ^1,3^), resulting in external quantum efficiencies (EQEs) of LED devices of less than 25%. Here we present a dual-additive crystallization method that enables the formation of highly efficient 3D perovskites, achieving an exceptional PLQE of 96%. This approach promotes the formation of tetragonal FAPbI_3_ perovskite, known for its high exciton binding energy, which effectively accelerates the radiative recombination. As a result, we achieve perovskite LEDs with a record peak EQE of 32.0%, with the efficiency remaining greater than 30.0% even at a high current density of 100 mA cm^−2^. These findings provide valuable insights for advancing the development of high-efficiency and high-brightness perovskite LEDs.

## Abstract (web summary)

A dual-additive crystallization method using PyNI and 5AVA as additives results in highly efficient 3D perovskite films with enhanced photoluminescence quantum efficiencies and external quantum efficiencies, and hence increased LED performance.

## Main

To achieve highly efficient and bright thin-film LEDs, a combination of factors is necessary for the light-emitting materials. These include high PLQE, high charge mobilities and low quantum efficiency droop. Traditional thin-film LEDs, such as organic LEDs, face challenges in achieving high efficiency at high brightness owing to their low charge mobility and susceptibility to Auger or excitonic quenching^7^. In recent studies, it has been demonstrated that low-dimensional perovskites with multiple-quantum-well or quantum dot structures, making use of quantum confinement effects, can effectively suppress nonradiative recombination^8–10^. This phenomenon allows them to achieve near 100% PLQE^9^. However, these low-dimensional perovskite materials often exhibit low charge mobility and suffer from severe Auger recombination, which limits their potential for efficient LEDs at high brightness^8,11^. On the other hand, 3D perovskites have emerged as promising materials for the development of efficient and bright thin-film LEDs. They have high charge mobility and show low quantum efficiency droop. Furthermore, 3D perovskites can naturally form discrete, sub-micrometre-scale structures, leading to an enhanced light outcoupling efficiency greater than 30% (ref. ^1^). Recent advancements have showcased large-sized, flexible, efficient and bright LEDs based on discrete 3D perovskites with excellent stability^12^. However, one challenge faced by 3D perovskites is their slow radiative recombination rate, making their PLQE highly susceptible to defects^4,13^. To address this issue, various passivation strategies have been used to reduce the defect density in 3D perovskite films, approaching levels comparable with single crystals^3^. Despite substantial efforts, the maximum achievable PLQE only reaches approximately 80%, with resulting LEDs exhibiting peak EQEs of less than 25% (refs. ^3,4^).

**Fig. 1:**
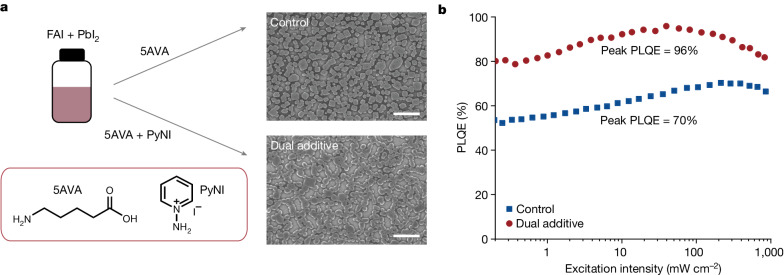
Fabrication process and characterization of perovskite films. **a**, Fabrication of perovskite films and SEM images of control perovskite (with 5AVA) and dual-additive perovskite (with 5AVA and PyNI). Scale bars, 1 µm. Chemical structures of 5AVA and PyNI. **b**, Excitation-intensity-dependent PLQEs of control and dual-additive perovskites. Peak PLQEs are about 70% and about 96% for control and dual-additive perovskites, respectively. Source Data

**Extended Data Fig. 1:**
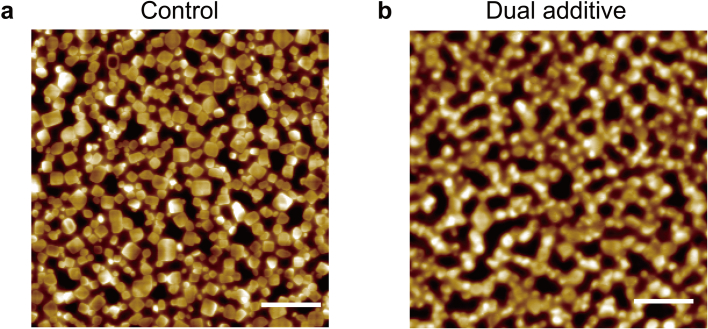
Atomic force microscopy images of perovskite films. **a**, Control perovskite. **b**, Dual-additive perovskite. Scale bars, 1 µm.

Here we demonstrate efficient, near-infrared 3D perovskites by accelerating the radiative recombination through formation of tetragonal FAPbI_3_ perovskite using a dual-additive method. The fabrication process is shown in Fig. 1a. A precursor solution was prepared using 1-aminopyridinium iodide (PyNI), 5-aminovaleric acid (5AVA), formamidinium iodide (FAI) and PbI_2_ with a molar ratio of 0.15/0.25/2.40/1.00 dissolved in *N,N*-dimethylformamide (DMF; 8.5 wt%) (see Methods for details), in which PyNI and 5AVA are both additives. For comparison, a control sample using only a single additive (5AVA) was also prepared^1^. We note that the role of 5AVA has been investigated previously, which can markedly enhance the LED efficiency by facilitating the formation of low-defect-density FAPbI_3_ perovskite with sub-micron discrete structures^1^. The control and dual-additive perovskites exhibit similar dispersed sub-micron grain morphologies (Fig. 1a and Extended Data Fig. 1), which can contribute to a light outcoupling efficiency greater than 30% in LED device architectures^1^ (Supplementary Note 1). Notably, the dual-additive sample shows a much higher PLQE of 96% compared with the control sample (about 70%) (Fig. 1b).

**Fig. 2:**
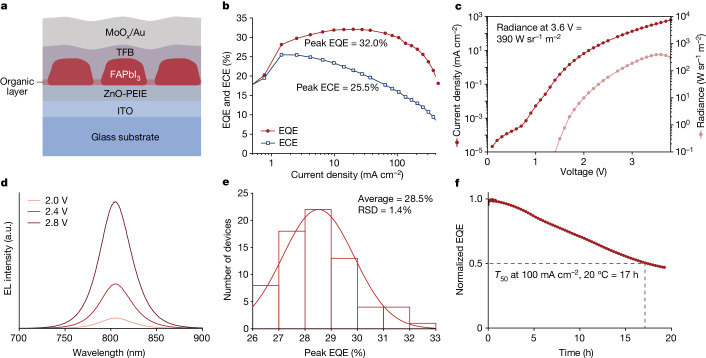
Device structure and performance of dual-additive perovskite LEDs. **a**, Schematic of the device structure. **b**, Current-density-dependent EQE and ECE. The dual-additive perovskite LED can achieve a peak EQE of 32% under a current density of 20 mA cm^−2^. **c**, Dependence of current density and radiance on bias voltage. The maximum radiance is 390 W sr^−1^ m^−2^ at 3.6 V. **d**, EL spectra at various voltages. **e**, Histogram of peak EQEs. Statistics from 70 devices show an average peak EQE of 28.5%, with a relative standard deviation (RSD) of 1.4%. **f**, Stability of the device measured at a constant current density of 100 mA cm^−2^ at 20 °C. a.u., arbitrary units. Source Data

**Extended Data Fig. 2:**
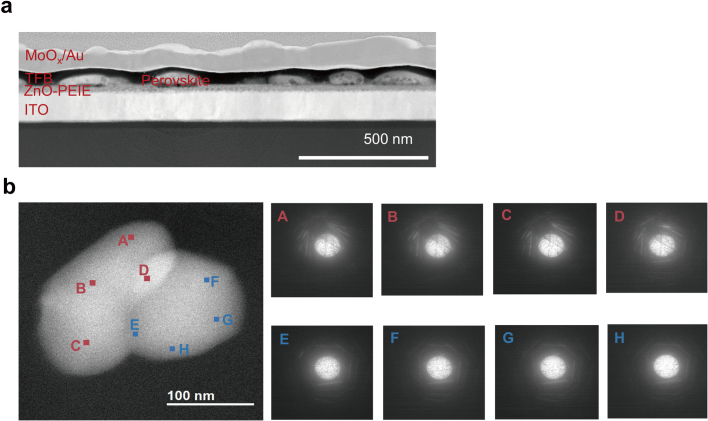
STEM images of dual-additive perovskite device and film. **a**, STEM cross-section image of the fabricated device. Scale bar, 500 nm. **b**, STEM images of the dual-additive perovskite film with Kikuchi line patterns in various regions. Scale bar, 100 nm.

**Extended Data Fig. 3:**
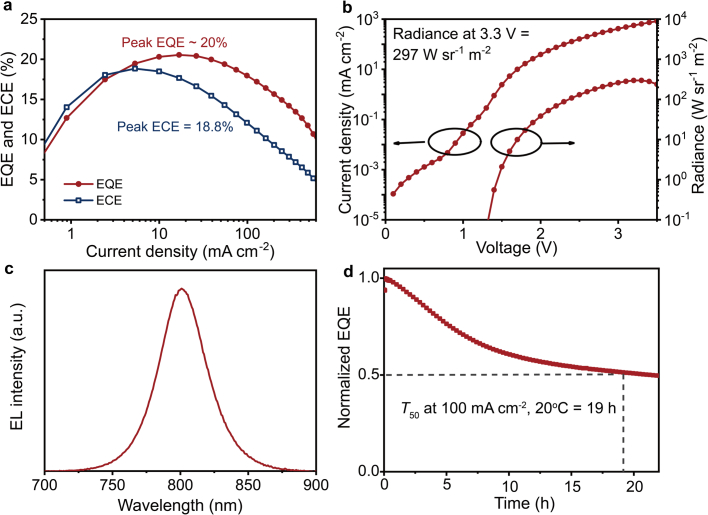
Performance of control perovskite LEDs. **a**, Current-density-dependent EQE and ECE. **b**, Dependence of current density and radiance on bias voltage (the maximum radiance is 297 W sr^−1^ m^−2^ at 3.3 V). **c**, EL spectrum. **d**, Stability of the device measured at a constant current density of 100 mA cm^−2^ at 20 °C. Source Data

Then we fabricated and characterized LEDs based on the two samples mentioned above. The device structure was indium tin oxide (ITO)/polyethylenimine ethoxylated (PEIE)-modified zinc oxide (ZnO; 30 nm)/perovskite (approximately 60 nm)/poly(9,9-dioctyl-fluorene-co-N-(4-butylphenyl)diphenylamine) (TFB; 20 nm)/molybdenum oxide (MoO_*x*_; 5 nm)/gold (Au; 80 nm)^1,3^ (Fig. 2a and Extended Data Fig. 2a). The dual-additive perovskite LEDs exhibit a peak EQE as high as 32.0% at a current density of 20 mA cm^−2^, accompanied by a maximum brightness of 390 W sr^−1^ m^−2^ at a low voltage of 3.6 V (Fig. 2b,c). The EQE remains high, with a value of 30% at a high current density of 100 mA cm^−2^. The peak energy conversion efficiency (ECE) reaches 25.5% at a current density of roughly 1 mA cm^−2^ and is maintained at 16.0% at a high current density of 100 mA cm^−2^ (Supplementary Note 2). The electroluminescence (EL) peak is located at 805 nm (Fig. 2d), which is slightly redshifted compared with the control device (801 nm) (Extended Data Fig. 3c). The devices demonstrate good reproducibility, with an EQE histogram for 70 devices showing an average peak EQE of 28.5% (Fig. 2e). By contrast, the control device exhibits a peak EQE of approximately 20% (Extended Data Fig. 3a). Considering they have similar light outcoupling efficiency (Supplementary Note 1), we believe that the improved performance of the dual-additive device can be mainly attributed to its higher PLQE (Fig. 1b). Also, we conducted measurement on the half-life (*T*_50_) stability of our devices under a constant current density of 100 mA cm^−2^. The *T*_50_ lifetimes of the devices were found to be comparable, with the control sample lasting for 19 h (Extended Data Fig. 3d), which aligns closely with the reported result^1^, and the dual-additive device lasting for 17 h (Fig. 2f). We believe that the slightly shorter lifetime of the dual-additive device could be because of the slightly more excess iodide ions with the dual-additive perovskite film, as the ion migration is the main cause of the device degradation^14^.

**Fig. 3:**
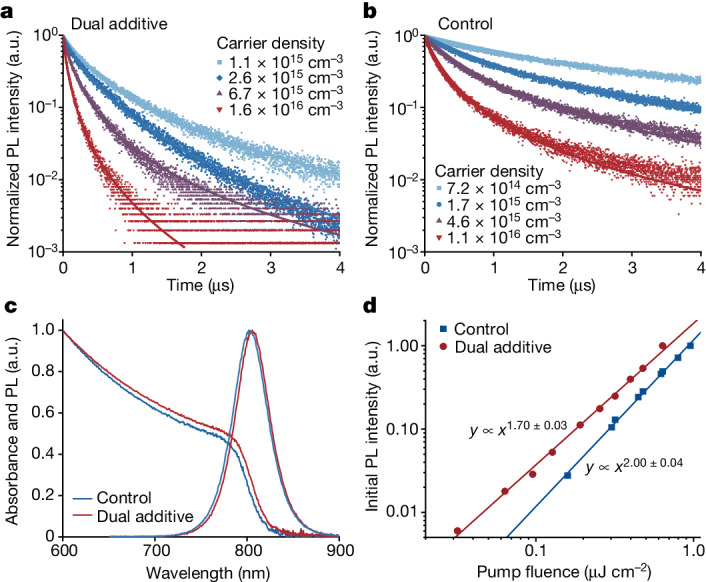
Optical properties of perovskite films. TRPL (scatter plots) under various excitation intensities and fitted by dynamics of charge-carrier models (curves) for dual-additive perovskite film (**a**) and control sample (**b**). **c**, Absorption and steady PL spectra of control and dual-additive perovskites. **d**, Logarithm plot of the integrated initial PL intensity (*I*_PL_[*t* = 0]) versus excitation density. a.u., arbitrary units. Source Data

**Extended Data Fig. 4:**
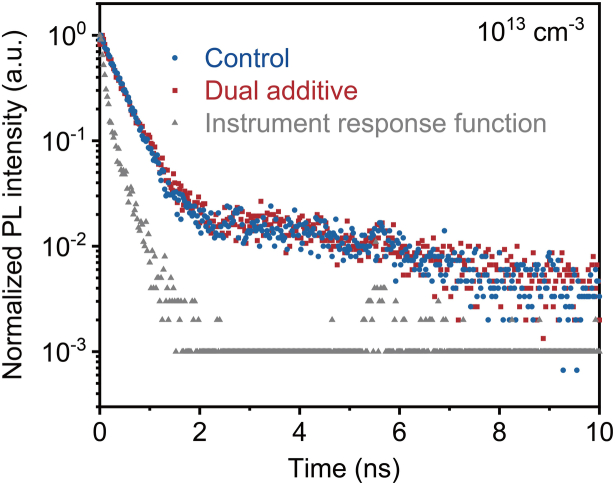
TRPL of perovskite films under extremely low excitation intensities. The excitation intensity is about 10^13^ cm^−3^. The dual-additive perovskite and control sample exhibit similar decay behaviour. Source Data

To verify why the dual-additive perovskite exhibits enhanced PLQE, we conducted time-resolved photoluminescence (TRPL) measurements under various excitation intensities. At an extremely low excitation intensity (with an initial carrier density of about 10^13^ cm^−3^), the TRPL of both the dual-additive perovskite and the control sample exhibit similar decay behaviour, indicating comparable trap densities between them^15^ (Extended Data Fig. 4). As the excitation intensities increase, the dual-additive perovskite exhibits accelerated photoluminescence (PL) decay compared with the control sample (Fig. 3a,b), which can be because of enhanced radiative recombination. To quantify this observation, we determined the first-order (trap-assisted and excitonic, *k*_1_) and second-order (bimolecular, *k*_2_) recombination rate constants for both samples by fitting the transient PL data, while keeping the third-order Auger recombination rate constant (*k*_3_)^4^ (Supplementary Note 3). The fitting results indicate that, regardless of the injected carrier density, the *k*_2_ value of the dual-additive perovskite surpasses that of the control sample by several folds, and *k*_1_ is approximately one order of magnitude higher for the dual-additive perovskite compared with the control sample (Extended Data Table 1). We found negligible effects on the fitting results by increasing/decreasing *k*_3_ for one order of magnitude (Supplementary Note 3). Because the trap densities between them are similar, the enhanced *k*_1_ in dual-additive perovskite is mainly because of the enhancement of excitonic (radiative) recombination instead of trap-assisted (nonradiative) recombination, which is consistent with the high PLQE observed in Fig. 1b under low excitation intensities. On the basis of these findings, we can conclude that the enhanced PLQE of the dual-additive perovskite film is associated with increased radiative recombination rates (both excitonic and bimolecular), rather than a decreased nonradiative recombination rate typically observed through defects passivation in previous studies^1–3^.

**Extended Data Fig. 5:**
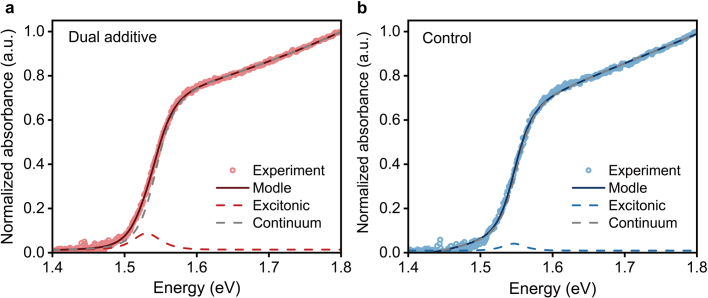
Fitting the curve of absorption spectra near to the band edge based on Elliott’s theory. **a**, Dual-additive perovskite. **b**, Control perovskite. The detailed fitting methods can be found in Supplementary Note 4. Source Data

We then investigate the underlying mechanism behind the enhanced radiative recombination rates. We observe that the absorption edge of the dual-additive perovskite film become more obvious (Fig. 3c), indicating a stronger excitonic feature compared with the control sample. By fitting the absorption spectra near the band edge based on Elliott’s theory^16^ (Supplementary Note 4), we obtain an exciton binding energy (*E*_b_) of 13.9 meV for the dual-additive perovskite film, which is much higher than that of the control sample (3.9 meV) (Extended Data Fig. 5). The increased *E*_b_ can lead to higher population of excitons in the excited states and enhance radiative recombination^17,18^, which is consistent with the fitting results (Extended Data Table 1). The more excitonic recombination can be further confirmed by power-dependent TRPL measurements at the zero time (*I*_PL_[*t* = 0]) (Fig. 3d). In these experiments, the PL intensities were measured immediately after the ultra-fast (fs) excitation, which can precisely determine the carrier-density-dependent PL intensity^13^. We then fit the PL intensities using a power-law function of the form PL ≈ *P*^*k*^, in which *P* represents excitation fluence and *k* is a real-number exponent providing information on the order of recombination. For dual-additive perovskite, a *k* value of 1.7 indicates the coexistence of free carriers (bimolecular recombination) and excitons (monomolecular recombination) (Supplementary Note 5). Conversely, *I*_PL_[*t* = 0] of the control perovskite increases quadratically (meaning a *k* value of 2) with the excitation fluence, suggesting predominantly bimolecular recombination. Therefore, we believe that the accelerated radiative recombination of the dual-additive perovskite is because of the increased *E*_b_, which is consistent with previous studies on the 3D FAPbI_3_ perovskite at low temperatures^19^.

**Fig. 4:**
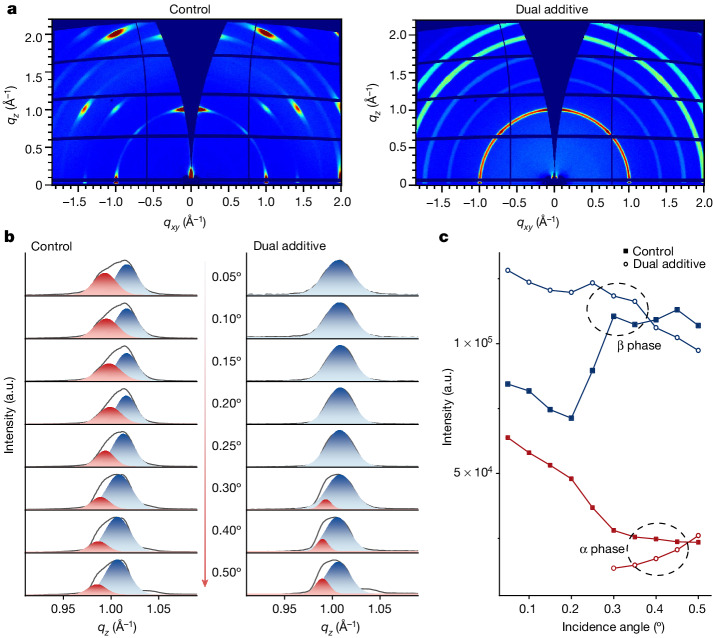
Structural characterization of perovskite films. **a**, 2D GIWAXS patterns of control and dual-additive perovskite films with the incidence angle of 0.20°. **b**, 1D GIWAXS patterns along *q*_*z*_ with various incidence angles. GIWAXS probing depth was varied by changing the angle of incidence of the X-ray beam from 0.05° to 0.50° (arrow indicates the incidence angles). Lower incidence angles imply a smaller probing depth in the perovskite surface, whereas larger angles indicate the detection of bulk perovskite. Owing to the distinct lattice spacings (*d*) between the cubic and tetragonal phases, measuring 6.40–6.35 Å and 6.34–6.30 Å, respectively, subtle variations in their corresponding *q* values were obtained^24^. The cubic phase (red) demonstrates a slightly smaller *q* value compared with the tetragonal phase (blue). **c**, Scattering intensity (peak area) obtained from the *q*_*z*_ fitting with various incidence angles. a.u., arbitrary units. Source Data

**Extended Data Fig. 6:**
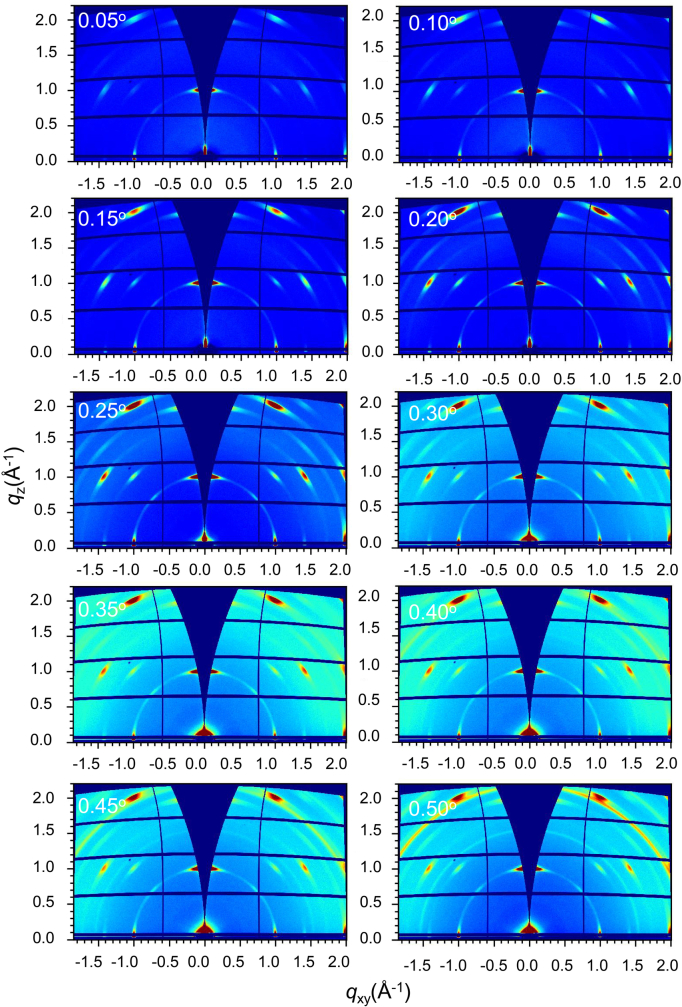
2D GIWAXS patterns of control perovskite film with various incidence angles. The incidence angles of 0.05°, 0.10°, 0.15°, 0.20°, 0.25°, 0.30°, 0.35°, 0.40°, 0.45° and 0.50° correspond to theoretical probing film depths of 5, 9, 80, 160, 210, 270, 320, 370, 420 and 480 nm, respectively.

**Extended Data Fig. 7:**
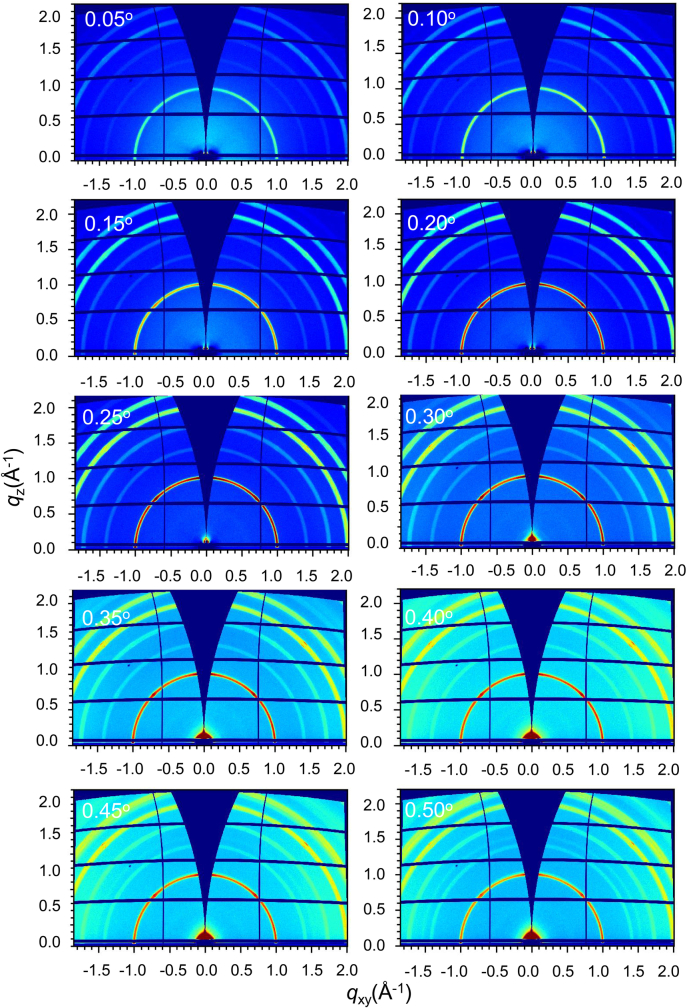
2D GIWAXS patterns of dual-additive perovskite film with various incidence angles. The incidence angles of 0.05°, 0.10°, 0.15°, 0.20°, 0.25°, 0.30°, 0.35°, 0.40°, 0.45° and 0.50° correspond to theoretical probing film depths of 5, 9, 80, 160, 210, 270, 320, 370, 420 and 480 nm, respectively.

The enhanced exciton binding energy in perovskites can result from increased space confinement of crystal grains or phase transitions^19–21^. In both the EL and PL spectra of the dual-additive perovskite, we observe an approximately 4 nm redshift compared with those of control sample (Figs. 2d and 3c and Extended Data Fig. 3c). This indicates that the dual-additive perovskite does not have enhanced space confinement. Otherwise, one would anticipate a blueshift in the emission. So, we can infer that a phase transition is responsible for the observed increase in binding energy. Also, because the dual-additive perovskite shows a similar trap density to the control sample, we believe that the trap-induced redshift does not occur in this case^22^. To investigate the crystal structure of the perovskite films, we conducted grazing-incidence wide-angle X-ray scattering (GIWAXS) analysis, varying the incidence angles (0.05° to 0.50°) to examine both the surface and bulk regions of the films. As shown in Fig. 4a, both the dual-additive perovskite and the control sample exhibit characteristic scattering at *q* ≈ 1.00 Å^−1^, which is attributed to the cubic or tetragonal perovskite^23,24^. Notably, the control sample shows the overlapping of discrete Bragg scattering spots and scattering rings (or arcs), whereas the dual-additive perovskite shows a scattering ring isotropic in polar angle. This result suggests a preferential stacking of vertical-oriented octahedrons along the surface-normal direction in the control sample, along with a small amount of random crystalline orientation (Extended Data Fig. 6), whereas the dual-additive perovskite has a random crystalline orientation (Extended Data Fig. 7). This observation is consistent with our morphology characterization that shows several types of grain packed in the dual-additive film (Extended Data Fig. 2b). We then analysed the *q*_*z*_ direction of 1D GIWAXS at various probing angles (or depths) (Fig. 4b). It shows that the control sample exhibits a uniform distribution of both cubic and tetragonal phases throughout the film. By contrast, the upper part of the dual-additive film is predominantly composed of a single tetragonal phase (0.05° to 0.25°), whereas the lower part exhibits mainly tetragonal phase with a small amount of cubic phases (Fig. 4c). It is consistent with the observed redshifted EL and PL spectra of the dual-additive perovskite film, as the tetragonal phase of FAPbI_3_ (refs. ^19,25^). On the basis of these investigations, we can conclude that the increased exciton binding energy in the dual-additive film is primarily attributed to the higher proportion of the tetragonal phase, which is consistent with the observation of the perovskite under low-temperature conditions^19,25^. Also, our transient PL measurement further shows that there is no substantial energy transfer occurring between the two phases (Supplementary Fig. 4), probably because of their very close bandgaps^25^ (Supplementary Note 6). Moreover, because the ZnO electron transport layer has a much higher charge mobility than the TFB hole transport layer^26,27^, we can expect that the electron–hole recombination mainly occurs near the interface of perovskite and TFB, which is predominantly of single tetragonal phase.

**Extended Data Fig. 8:**
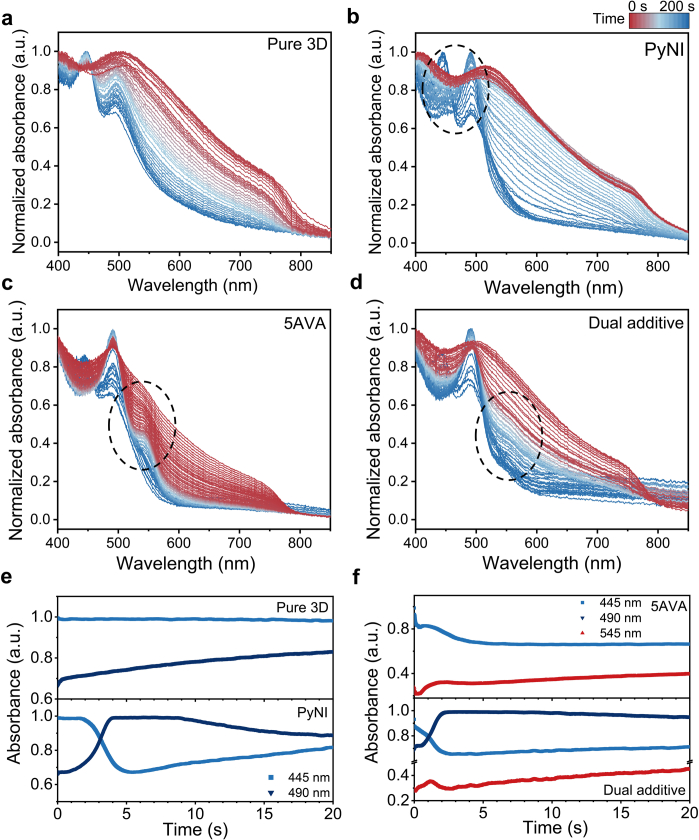
Annealing-time-dependent absorption spectra of FAPbI_3_ perovskites with various additives. **a**, Pure 3D perovskite without additive. **b**, PyNI-based perovskite. **c**, 5AVA-based perovskite. **d**, Dual-additive perovskite. **e**, Annealing-time-dependent absorption changes of pure 3D and PyNI-based perovskite at 445 and 490 nm. **f**, Annealing-time-dependent absorption changes of 5AVA-based perovskite and dual-additive perovskite at 445, 490 and 545 nm. Source Data

To investigate how the dual additives can induce an increased proportion of the tetragonal phase, we conducted in situ absorption-spectra measurements during the film-annealing process (Extended Data Fig. 8). We observe that PyNI-additive-alone perovskite film exhibits dominant absorption peaks at 445 nm (PbI_4_^2−^) and 490 nm (PbI_6_^4−^), similar to the samples without additive or with 5AVA alone^28^. Notably, the PyNI-additive-alone and dual-additive films show a rapid decrease in absorption at 445 nm, along with an increase at 490 nm, indicating that PyNI can facilitate the conversion of PbI_4_^2−^ to PbI_6_^4−^ during annealing. In the cases of the 5AVA-alone and dual-additive films, we observe a further absorption peak at 545 nm, which can be attributed to the formation of a 5AVA-related low-dimensional intermediate phase^3^. These results indicate the coexistence of two crystallization pathways in the dual-additive system induced by the two additives separately. Previous studies have shown that the competition between these two crystallization pathways can lead to the confinement of the crystals, resulting in a higher proportion of the tetragonal phase^29,30^. Notably, from scanning transmission electron microscopy (STEM) measurements, we can observe the variation in Kikuchi line patterns among various regions within each particle for the dual-additive perovskite (Extended Data Fig. 2b). This suggests that the particles in dual-additive perovskite consist of several grains that are tightly connected or interlocked. We believe that our result of the formation of more tetragonal phase in dual-additive perovskite is consistent with previous studies.

To achieve high-efficiency LEDs with superior brightness, it is crucial to use light-emitting materials that exhibit high PLQEs, minimal Auger or excitonic quenching, high charge mobilities and preferably have structures conducive to efficient light outcoupling. Existing thin-film light-emitting materials, including organic semiconductors, quantum dots and low-dimensional perovskites, have fallen short of meeting all these criteria simultaneously. In our study, we present a straightforward approach to address this challenge by using 3D perovskites with increased exciton binding energy, which facilitate an accelerated rate of radiative recombination. By promoting the formation of tetragonal FAPbI_3_ perovskite, we have successfully achieved a near-unity PLQE in the 3D perovskite film. This remarkable achievement has enabled us to realize LEDs with an unprecedented EQE record of 32.0%. Our work holds pivotal importance in paving the way for continuous advancements in breaking the efficiency limits of perovskite LEDs and unlocks their full capabilities in next-generation display and lighting technologies.

## Methods

### Perovskite precursor solution

The dual-additive perovskite precursor solution was prepared by dissolving PyNI, 5AVA, FAI and PbI_2_ with a molar ratio of 0.15/0.25/2.40/1.00 at 8.5 wt% in DMF (Supplementary Note 7). For the control sample, 5AVA, FAI and PbI_2_ were dissolved with a molar ratio of 0.7/2.4/1.0 at 7 wt% in DMF. The prepared solution was stirred in a N_2_ glovebox over 2 h before being used as precursor.

### Film and device fabrication

After washing by ethanol, ITO glass substrates were dried with nitrogen gas and subsequently treated by oxygen plasma (70 W) for 10 min. ZnO nanocrystals (Supplementary Note 7) were deposited onto the ITO glass by spin coating (4,000 rpm for 45 s) and annealed at 150 °C for 30 min in air. Subsequently, a PEIE layer (3 mg ml^−1^ in 2-methoxyethanol) was deposited onto the ZnO layer at 5,000 rpm for 50 s, followed by annealing at 100 °C for 10 min in air. Perovskite film was deposited onto the PEIE-treated ZnO film by spin coating (4,000 rpm for 45 s) and annealed at 105 °C for 18 min in a N_2_ glovebox. TFB layer was deposited onto the perovskite film by spin coating (2,000 rpm for 45 s) from solution (10 mg ml^−1^ in m-xylene). Finally, 5 nm of MoO_*x*_ and 80 nm of Au layers were subsequently thermal evaporated as electrodes through a shadow mask at a rotation speed of 12 rpm at a pressure of 1 × 10^−5^ Pa. The evaporation rate for both layers was 0.1 Å s^−1^. The device area was defined by the overlap area of the ITO film and Au electrode, which was 3 mm^2^.

### Device characterization

The perovskite LED devices were measured in a glovebox at room temperature using a combination of a fibre integration sphere (FOIS-1) coupled with a QE65 Pro spectrometer and a Keithley 2400 source meter^1,8^. The LEDs were mounted on top of the integration sphere and only forward light can be collected. The devices were subjected to a voltage sweep from 0 to 5 V (which may stop earlier subject to the point of reaching maximum brightness) at a rate of 0.1 V s^−1^. Details on efficiency measurement and calculation can be found in Supplementary Note 2. We measured the angular dependence of spectra using a QE65 Pro spectrometer. The characterization system was cross-checked between three different labs to ensure accuracy in the measurements^1^ (Supplementary Note 2). Stability measurement was carried out using a Keithley 2450 source meter, a Keithley 2000 electric meter and a photodetector (Thorlabs PDA100A). The devices were operated at a constant current density of 100 mA cm^−2^ at 20 °C in a N_2_ glovebox.

### Film characterization

The steady-state absorption spectra were measured by an ultraviolet–visible spectrophotometer with an integrating sphere (PerkinElmer, Lambda 950). The steady-state photoluminescence spectra were measured using a QE65 Pro spectrometer and a 445-nm continuous-wave laser as an excitation source. In situ absorption spectra were measured by an ISAS-HI001 system (Nanjing Ouyi Optoelectronics Technology). An Ocean Optics HL-2000 was used as a white light source. We collected the morphology of perovskite films by scanning electron microscopy (SEM; JEOL5 JSM-7800F) and atomic force microscopy (MutilMode 8, Bruker). The PLQEs of perovskite films were measured by combing a 445-nm continuous-wave laser with excitation intensities ranging from 0.1 to 1,000 mW cm^−2^, two optical fibres, a spectrometer and an integrating sphere. TRPL measurements were performed by a combination of a single photon counting module (COUNT-100T-FC, Laser Components GmbH), a TimeHarp 260 PICO board (PicoQuant), two monochromators (iHR320, HORIBA) and a pulsed white laser (6 ps, 0.1 MHz, Fianium WhiteLaseSC400 High Power Supercontinuum). The excitation for TRPL measurement was 650 nm.

For the zero-time PL (*I*_PL_[*t* = 0]) measurement, the femtosecond laser source was a Coherent Astrella-1K-F Ultrafast Ti:Sapphire Amplifier with a pulse duration of 100 fs, repetition rate of 1 kHz and wavelength of 800 nm. This amplifier was seeded by a Coherent Vitesse oscillator. The 650-nm laser used in the experiment was obtained from the optical parametric amplifier configuration. TRPL spectra were acquired using a Hamamatsu streak camera system with an ultimate temporal resolution of 1 ps.

### STEM measurement

For the preparation of perovskite film samples for STEM measurement, we use a polymethyl methacrylate (PMMA) layer to transfer the perovskite film onto a copper grid. Typically, PMMA was spin coated (*V*_PMMA_/*V*_CB_ = 1/1) onto cleaned ITO glass at 4,000 rpm for 60 s. The PMMA sample was then transferred to a 200 °C hotplate and annealed for 20 min, followed by plasma treatment for 1 min. Then ZnO/PEIE/perovskite layers were spin coated onto the PMMA layer using the aforementioned method. The fabricated sample was then placed in dichloromethane solvent for 10 s, which can dissolve the PMMA layer. Consequently, the ZnO/PEIE/perovskite layers were separated from the substrates and can be collected by a copper grid for STEM measurement after drying.

For preparation of the cross-sectional device sample for STEM measurement, a focused-ion-beam system (FEI Helios 600i) was used. The microstructure is characterized using aberration-corrected STEM at high-angle annular dark-field mode on a FEI Titan G2 80-200 microscope at an emission voltage of 200 kV, equipped with a Super-X EDX detector.

### GIWAXS measurement

The preparation of GIWAXS samples is consistent with the above film fabrication method, with a structure of ITO/ZnO/PEIE/perovskite. The fabricated samples are encapsulated in a N_2_ atmosphere before measurement. The GIWAXS experiments were carried out at beamline BL17B1 at the Shanghai Synchrotron Radiation Facility (SSRF). The data were obtained with a PILATUS detector of 1,475 × 1,679 pixels resolution (253.7 × 288.18 mm). The monochromated energy of the X-ray source was about 10 keV, with a high energy resolution of approximately 10^−4^. The X-ray wavelength was 1.2378 Å and the incidence angle was optimized between 0.05° and 0.50°. The 2D GIWAXS patterns were analysed using the FIT 2D software and shown in scattering vector *q* coordinates.

## Online content

Any methods, additional references, Nature Portfolio reporting summaries, source data, extended data, supplementary information, acknowledgements, peer review information; details of author contributions and competing interests; and statements of data and code availability are available at 10.1038/s41586-024-07460-7.

### Supplementary information

Supplementary Information Supplementary Notes 1–7 including Supplementary Figs 1–5 and Supplementary Tables 1 and 2.

Source data for Supplementary Figs 2–5 .

### Source data

Source Data Fig. 1

Source Data Fig. 2

Source Data Fig. 3

Source Data Fig. 4

Source Data Extended Data Fig. 3

Source Data Extended Data Fig. 4

Source Data Extended Data Fig. 5

Source Data Extended Data Fig. 8

## Figures and Tables

**Extended Data Table 1:**
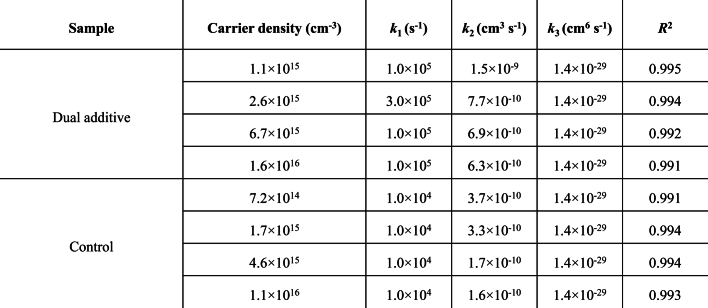
Summary of the fitting results of charge-carrier recombination rate constants

The first-order recombination rate constants (trap-assisted and excitonic, *k*_1_), second-order recombination rate constants (bimolecular, *k*_2_), third-order Auger recombination rate constants (*k*_3_) and the *R*^2^ of the fitting are presented. The detailed fitting methods can be found in Supplementary Note 3.

## Data Availability

The data supporting the findings of this study are fully and freely available from the corresponding author. Source data are provided with this paper.
