# Author Correction: Acceleration of radiative recombination for efficient perovskite LEDs

**DOI:** 10.1038/s41586-024-07665-w

**Published:** 2024-06-13

**Authors:** Mengmeng Li, Yingguo Yang, Zhiyuan Kuang, Chenjie Hao, Saixue Wang, Feiyue Lu, Zhongran Liu, Jinglong Liu, Lingjiao Zeng, Yuxiao Cai, Yulin Mao, Jingshu Guo, He Tian, Guichuan Xing, Yu Cao, Chao Ma, Nana Wang, Qiming Peng, Lin Zhu, Wei Huang, Jianpu Wang

**Affiliations:** 1 Key Laboratory of Flexible Electronics (KLOFE), Institute of Advanced Materials (IAM) & School of Flexible Electronics (Future Technologies), Nanjing Tech University, Nanjing, China; 2 Strait Institute of Flexible Electronics (SIFE, Future Technologies), Fujian Normal University, Fuzhou, China; 3 School of Microelectronics, Fudan University, Shanghai, China; 4 Center of Electron Microscopy, State Key Laboratory of Silicon Materials, School of Materials Science and Engineering, Zhejiang University, Hangzhou, China; 5 Institute of Applied Physics and Materials Engineering, University of Macau, Macau, China; 6 State Key Laboratory of Extreme Photonics and Instrumentation, College of Optical Science and Engineering, International Research Center for Advanced Photonics, Zhejiang University, Hangzhou, China; 7 Strait Laboratory of Flexible Electronics (SLoFE), Fuzhou, China; 8 Institute of Flexible Electronics (IFE), Northwestern Polytechnical University (NPU), Xi’an, China; 9 MIIT Key Laboratory of Flexible Electronics (KLoFE), Northwestern Polytechnical University (NPU), Xi’an, China; 10 School of Flexible Electronics (SoFE), Sun Yat-sen University, Shenzhen, China; 11 School of Materials Science and Engineering, Changzhou University, Changzhou, China; 12 School of Microelectronics and Control Engineering, Changzhou University, Changzhou, China

**Keywords:** Lasers, LEDs and light sources, Materials for devices

Correction to: *Nature* 10.1038/s41586-024-07460-7Published online 29 May 2024

In the version of the article initially published, a note of thanks to Dr. Gang Wang was missing from the Acknowledgements section.

