# Supplementary material for: Acceleration of radiative recombination for efficient perovskite LEDs
Source: Nature. 2024 May 29;630(8017):631–5. doi: 10.1038/s41586-024-07460-7 (PMC11186751; doi:10.1038/s41586-024-07460-7)
Supplement: Supplementary file 1 — Supplementary Notes 1–7 including Supplementary Figs 1–5 and Supplementary Tables 1 and 2. [file 41586_2024_7460_MOESM1_ESM.pdf]

nature portfolio

https://doi.org/10.1038/s41586-024-07460-7

Supplementary information

Acceleration of radiative recombination for efficient perovskite LEDs

In the format provided by the authors and unedited

Table of Contents

Supplementary Note 1: Optical simulations

Supplementary Fig. 1. Analysis of frequency spectrum of the randomly distributed perovskite map by using two-dimensional FFT

Supplementary Note 2: Device characterization and cross-checking of EQE measurement system

Supplementary Fig. 2. The angular distribution of the radiation intensity of dual-additive perovskite LED follows a Lambertian profile

Supplementary Note 3: TRPL fitting

Supplementary Fig. 3. TRPL data fitting with various Auger rate constants for dual-additive perovskite

Supplementary Table 1 Summary of fitting result of charge-carrier recombination rate constants with various Auger rate constants or k_1 (dual-additive perovskite)

Supplementary Note 4: Exciton binding energy (E_b) calculation

Supplementary Note 5

Supplementary Fig. 4. Transient PL spectra of control perovskite at various time delays

Supplementary Note 6

Supplementary Fig. 5. The electronic band structure diagrams for cubic and tetragonal phases of $FAPbI_3$

Supplementary Note 7

Supplementary Table 2 Summary of materials and their sources

Supplementary Note 1:

Optical simulations. We conducted 3D Finite-Difference-Time-Domain (FDTD) simulations to calculate the outcoupling efficiency, following the methodology developed in our prior publication[1]. And we have shown that the outcoupling efficiency of the control device is ~30%[1]. A basic assumption is that the outcoupling efficiency of the device with randomly distributed perovskite pattern can be estimated by those reference devices with regularly distributed perovskite pattern and similar periodicity. Initially, we examined the spatial frequency spectrum of the randomly distributed perovskite pattern through a two-dimensional Fast Fourier Transform (FFT) analysis. The predominant spatial frequency components provided us with the range of periods to choose the reference devices with regularly distributed perovskite pattern. Upon discretizing the SEM data and applying FFT, we determined the perovskite periods to fall within the range of 330-1100 nm (Supplementary Fig. 1). Subsequently, we employed commercial software (Lumerical FDTD) to perform FDTD simulations for the reference devices with corresponding period, using a duty cycle of 50%. The device structure included an 80-nm-thick metal layer (Au), a 5-nm-thick MoO_x layer, a 20-nm-thick TFB layer, a 60-nm-thick perovskite emitting layer, a 30-nm-thick layer of ZnO-PEIE, a 100-nm-thick ITO layer, and a semi-infinite glass substrate. The refractive indices assigned to different layers in the simulation were consistent with those previously reported in our research and determined using an ellipsometer. The near field at the glass-ITO interface was utilized to calculate the outcoupled far field in air through the far field analysis group in the software with the reflection in the glass-air interface considered. To represent the incoherent isotropic light generation process, the simulation was repeated with three different source polarizations to obtain a weighted result. Besides, the multiple runs of the above operation were carried with adjusted light-emitting source location, whose results were weighted to extract the outcoupling efficiency of the reference device finally. Ultimately, the calculated outcoupling efficiency for our dual-additive perovskite LED devices was determined to be 32.09% ± 5.66%, which is close to that of the control device.

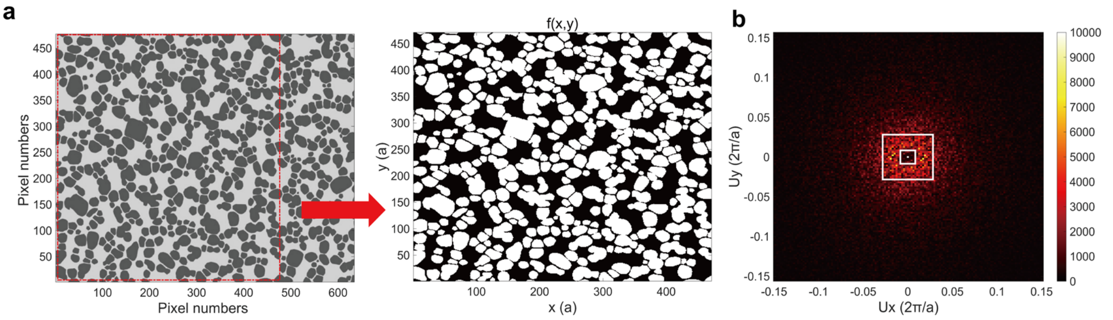

Supplementary Fig. 1 Analysis of frequency spectrum of the randomly distributed perovskite map by using two-dimensional FFT. a, Discretized map of the perovskite layer. x and y are the pixel numbers in units of pixel length a. $f(x,y)$ is the discrete function. **b**, Module of spatial frequency spectrum. U_x and U_y are the spatial frequencies.

Supplementary Note 2:

Device characterization and cross-checking of EQE measurement system.

We use an integrating sphere to get the absolute intensity of the forward light ($P(\lambda)$) emitted from device surface. The current through the device is recorded by a Keithley 2400 source meter. Then, the EQE and ECE can be calculated by the following Eq. 1 and Eq. 2, respectively.

$$\mathrm{EQE} = \frac{N_{photon}}{N_{electron}} = \frac{\int \frac{P(\lambda)\, d\lambda}{h\nu(\lambda)/t}}{I \times t/e} = \frac{e}{I \times h} \int \frac{P(\lambda)}{\nu(\lambda)} d\lambda \quad \text{(Eq. 1)}$$

$$\mathrm{ECE} = \frac{\int P(\lambda)\, d\lambda}{IV} \quad \text{(Eq. 2)}$$

For a Lambertian surface (Supplementary Fig. 2), the light intensity $P(\lambda)$ can be calculated by Eq. 3.

$$P(\lambda) = \int \mathrm{dS} \int_{\Omega} L(\lambda) \cos\theta \, d\Omega = S \int_{0}^{\pi/2} L(\lambda) \cos\theta \sin\theta d\,\theta = \pi S L(\lambda) \quad \text{(Eq. 3)}$$

Therefore, the radiance (L) can be calculated by Eq. 4.

$$L = \int L(\lambda)\, d\lambda = \frac{\int P(\lambda)\, d\lambda}{\pi S} \quad \text{(Eq. 4)}$$

where λ represents the device emission wavelength, h represents Planck's constant, e represents single electron charge, v represents photon frequency, t represents spectral integration time, V represents the applied voltage across the device, I represents the applied current across the device, S represents the device area, Ω is the solid-angle in the direction of emission, θ is the angle between the normal of the surface and the direction of the emission.

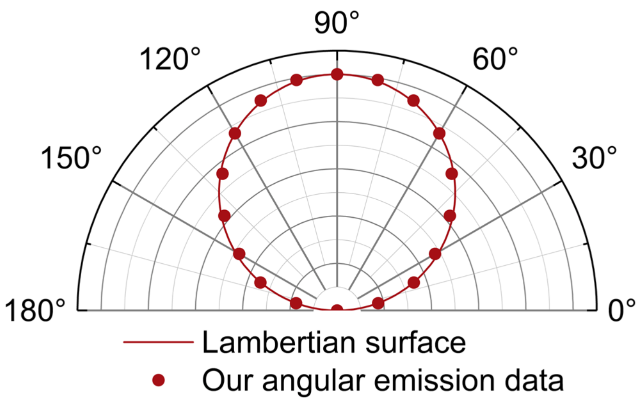

Supplementary Fig. 2 Angular distribution of the radiation intensity of our dual-additive perovskite LED. The measured angular distribution of the radiation intensity of dual-additive perovskite LED follows a Lambertian profile.

To ensure the reliability of the data, we have cross-checked the EQE measurement method with other two labs, the Optoelectronics Group of Cambridge University (UK) and professor Yizheng Jin group of Zhejiang University (China). Specifically, same batch of perovskite LED devices were prepared at Nanjing Tech University, which were divided to three sets and measured simultaneously when the Cambridge group received its set. As for the measurement methods, the Nanjing Tech and Zhejiang University groups used the same technique, as mentioned above[1–3]. While the Cambridge group used a combination of calibrated silicon photodetector and a Keithley 2400 source meter[4,5] to collect all forward photons. Importantly, the measurement results from the three groups are in good agreement, demonstrating the reliability of the measurement systems[1].

Supplementary Note 3:

TRPL fitting. To obtain the trap density, the TRPL spectra were fitted by a generic kinetic model proposed by Samuel et al.[6] We have established a simple model to describe the dynamics of charge carriers in the thin film, as shown in the following Eq. 5.

$$\frac{dn(t)}{dt} = -k_1 n(t) - k_2 n(t)^2 - k_3 n(t)^3 \quad \text{(Eq. 5)}$$

where n(t) is the carrier density as a function of time t, k_1, k_2, and k_3 are the rate constants of trap-assisted, bimolecular and Auger recombination, respectively. Through this model, the TRPL curves at various excitation intensities are fitted simultaneously using a global algorithm, which is called as Simulated Annealing[7]. The initial carrier density $n(0)$ is set as the boundary condition for the fitting. In calculating $n(0)$, the absorption of the film was considered. The values of k_1, k_2, and k_3 obtained are all the most fitting results after 100 iterations of the Simulated Annealing algorithm.

The Auger recombination rate constant was adopted from literature[8]. Importantly, we find that the Auger recombination has a negligible effect on the carrier dynamics in our study. For example, at a high carrier density of 1×10^{16} cm^{-3} and assuming the recombination rate constants as k_1 = 10^5 s^{-1}, k_2 = 6×10^{-10} cm^3 s^{-1}, and k_3 = 1.4×10^{-29} cm^6 s^{-1}, the monomolecular and bimolecular recombination rates are 1×10^{21} and 6×10^{22} cm^{-3} s^{-1}, respectively. While, the Auger recombination rate is merely 1.4×10^{19} cm^{-3} s^{-1}, which is significantly lower by 2-3 orders of magnitude compared to the other terms. Moreover, we have conducted additional calculations to evaluate the impact of Auger recombination on the carrier dynamics by increasing/decreasing k_3 for one order of magnitude, and found negligible effects on the fitting results (Supplementary Fig. 3 and Supplementary Table 1).

It is noteworthy that the increase in k_1 at a carrier density of 2.6×10^{15} cm^{-3} might be within the fitting error. We have refitted k_1 as either 1.0×10^5 or 1.5×10^5 s^{-1}, resulting in R^2 values of 0.991 and 0.992, respectively (Supplementary Table 1).

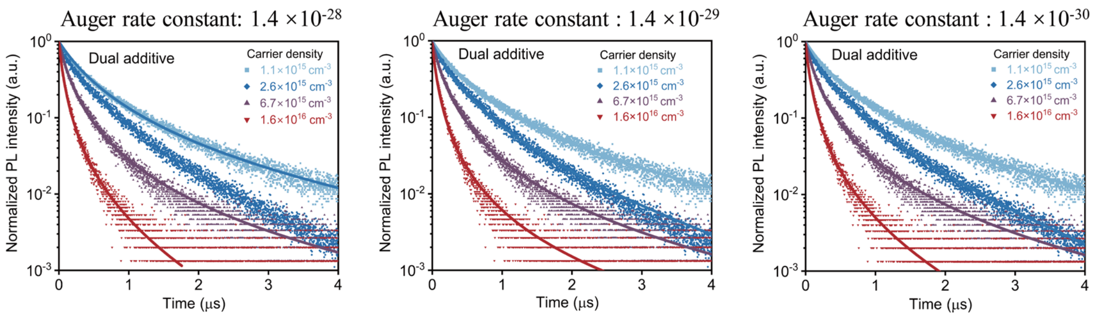

Supplementary Fig. 3 TRPL data fitting with various Auger rate constants for dual-additive perovskite. The Auger rate constants are as indicated, which have negligible effects on the fitting results.

Supplementary Table 1 Summary of fitting result of charge-carrier recombination rate constants with various Auger rate constants or k_1 (dual-additive perovskite). The first-order recombination rate constants (trap-assisted and excitonic, k_1), second-order recombination rate constants (bimolecular, k_2), third-order Auger recombination rate constants (k_3), and the R^2 of the fitting are presented.

Carrier density (cm^{-3})	k_1 (s^{-1})	k_2 ($cm^3\ s^{-1}$)	k_3 ($cm^6\ s^{-1}$)	R^2
1.1×10^{15}	1.0×10^5	1.4×10^{-9}	1.4×10^{-30}	0.995
	1.0×10^5	1.5×10^{-9}	1.4×10^{-29}	0.995
	1.0×10^5	1.4×10^{-9}	1.4×10^{-28}	0.995
2.6×10^{15}	3.2×10^5	7.2×10^{-10}	1.4×10^{-30}	0.995
	3.0×10^5	7.7×10^{-10}	1.4×10^{-29}	0.994
	1.0×10^5	9.2×10^{-10}	1.4×10^{-29}	0.991
	1.5×10^5	8.6×10^{-10}	1.4×10^{-29}	0.992
	3.1×10^5	7.4×10^{-10}	1.4×10^{-28}	0.995
6.7×10^{15}	1.1×10^5	6.9×10^{-10}	1.4×10^{-30}	0.993
	1.0×10^5	6.9×10^{-10}	1.4×10^{-29}	0.992
	1.1×10^5	6.9×10^{-10}	1.4×10^{-28}	0.993
1.6×10^{16}	1.0×10^5	6.4×10^{-10}	1.4×10^{-30}	0.992
	1.0×10^5	6.3×10^{-10}	1.4×10^{-29}	0.991
	1.0×10^5	6.3×10^{-10}	1.4×10^{-28}	0.992

Supplementary Note 4:

Exciton binding energy (E_b) calculation. We use the Elliott formula to fit the absorption spectrum[9]. According to the formula, the relationship between absorption coefficient and photon energy can be described as below:

$$\alpha(\hbar\omega) \propto [\sum_i \frac{2E_b}{i^3} sech(\frac{\hbar\omega-(E_g-\frac{E_b}{i^2})}{\Gamma}) + A\times arctan(sinh(\frac{\hbar\omega-E_g}{\Gamma}))(1-exp(-2\pi(\frac{E_b}{|\hbar\omega-E_g|})^{0.5}))^{-1}(1-B(\hbar\omega-E_g))^{-1}] \quad \text{(Eq. 6)}$$

where, E_b and E_g are the exciton binding energy and bandgap of a material. Γ refers to the spectral broadening. A and B are both constant. The first part of the formula donates the exciton contribution term and the second donates the continuum term. Due to the different E_b dependence of two terms, the value of E_b can be determined by fitting the curve of absorption spectrum near to the band edge.

Supplementary Note 5:

For the k values in Fig. 3d, we note that the initial PL intensity (PL_0) is directly proportional to the number of photons emitted per unit time at t=0, which can be expressed as:

$$PL_0 = k_r n + k_2 n^2 \qquad \text{(Eq. 7)}$$

where k_r and k_2 represent the exciton recombination rate and free carrier recombination rate, respectively, n represents the initial injected carrier density. When bimolecular recombination dominates, the first term in Eq. 7 can be neglected, and a k value of 2 can be obtained. When carriers exclusively recombine in excitonic recombination dominates, the second term in Eq. 7 can be neglected, and thus a k value of 1 should be expected. For the existence of both excitonic and bimolecular recombination processes, we can express Eq. 7 in logarithmic form:

$$\ln PL_0 = \ln(k_r n + k_2 n^2) \qquad \text{(Eq. 8)}$$

To fit the PL intensity at time zero as a function of excitation density, we employ the following equation.

$$PL_0 = \alpha n^k \qquad \text{(Eq. 9)}$$

where α is a proportionality parameter. To obtain a first-order linear approximation of Eq. 8, a Taylor expansion is performed and we have the expression for the k value in Eq. 9 as:

$$k = 1 + \frac{k_2 n^2}{k_r n + k_2 n^2} \qquad \text{(Eq. 10)}$$

Therefore, a k value of 1~2 implies that both the excitonic and bimolecular recombination processes should be considered.

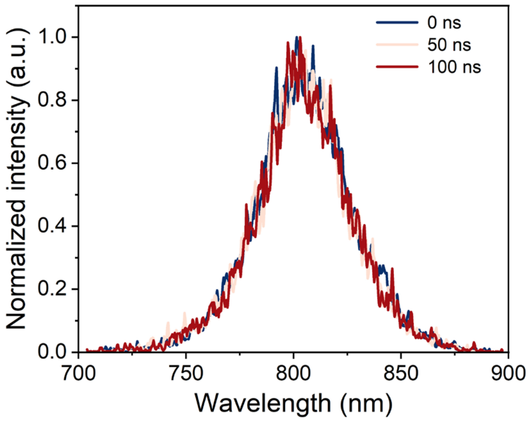

Supplementary Fig. 4 Transient PL spectra of control perovskite at various time delays. Transient photoluminescence data is acquired using an EMICCD (PI-MAX4 512B). Fianium WhiteLase SC400 white laser with 0.2 MHz 6 ps pulse was used for excitation.

Supplementary Note 6:

We conducted the theoretical calculations to investigate the cubic and tetragonal phases, and found a minimal difference in the band gap between them (Supplementary Fig. 5), which is consistent with the literature[10]. The initial structure of the cubic phase of $FAPbI_3$ was acquired from the CIF file reported in the previous study[11]. While for the tetragonal phase, no corresponding CIF file was available. Consequently, Cs^+ was directly substituted for FA^+ in the tetragonal $CsPbI_3$ phase[12]. Subsequently, the resulting structure underwent relaxation through DFT calculations to achieve optimal stability. According to the usually inaccurate position of light element, we only apply structure relaxation calculation on FA^+ while the Pb^{2+} and I^- remain still.

The structural relaxation and band structure calculations were conducted using a plane-wave pseudopotential approach implemented in the DS-PAW package[13]. The PBE functional was employed to account for exchange-correlation effects. The plane-wave cutoff energy was set at 300.0 eV. For the cubic phase, a k-point grid of 5 × 5 × 5 was used, while for the tetragonal phase, a grid of 4 × 4 × 5 was applied. Due to the heavy atom contained in perovskite materials, spin orbit coupling is included in DFT calculation for describing the real physical scenario.

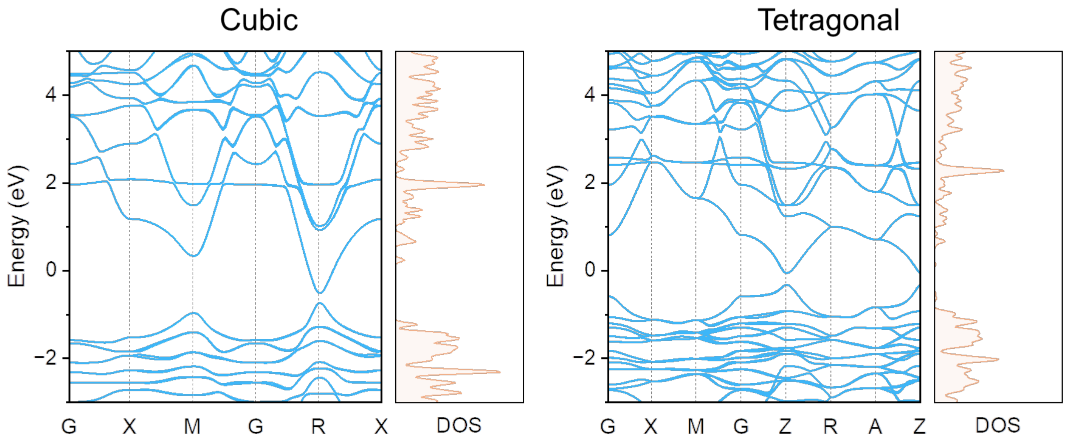

Supplementary Fig. 5 The electronic band structure diagrams for cubic and tetragonal phases of $FAPbI_3$. The results indicate a minimal difference in the band gap between the samples.

Supplementary Note 7:

Materials. All the chemicals were used as received, as detailed in Supplementary Table 2.

Supplementary Table 2 Summary of materials and their sources. The first column lists the material names, with additional information such as purity or molecular weight enclosed in parentheses. The second column indicates the company from which the materials were purchased.

Materials	Sources
1-Aminopyridinium Iodide (PyNI, >98%)	TCI
5-Aminovaleric acid (5AVA, 97%) and dimethyl sulfoxide (DMSO, 99.8%)	Aladdin
Formamidinium iodide (FAI, ⩾99.5%)	Xi'an Yuri Solar
PbI_2 (99.9985%) and MoO_x (99.95%)	Alfa Aesar
N,*N*-Dimethylformamide (DMF, anhydrous, 99.8%), PEIE (37 wt.% in H_2O), tetramethylammonium hydroxide pentahydrate (TMAH, ⩾95.0%), zinc acetate hydrate, and 2-methoxyethanol (anhydrous, 99.8%)	Sigma-Aldrich
M-xylene (99%) and Ethanol (99.7%)	J&K Scientific
Ethanolamine (99%)	Thermo Scientific
Poly(9,9-dioctylfluorene-co-*N*-(4-butylphenyl)diphenylamine) (TFB, molecular weight >30,000)	American Dye Source
Polymethyl methacrylate (PMMA)	Microchem
Chlorobenzene (99%)	Honeywell
Dichloromethane (HPLC, ⩾99.9%)	Innochem
Copper grid (BZ11032a, mesh:200)	Zhongjingkeyi Films Technology Co.,Ltd

Synthesis of ZnO nanocrystals. Synthesis of ZnO nanocrystals was followed our previous studies[1,2,14,15]. An ethanol solution (10 mL) of TMAH (5.6 mmol) was added dropwise to a dimethyl sulfoxide solution (30 mL) of zinc acetate hydrate (3 mmol) and stirred at 300 rpm for 24 h at 30°C. ZnO nanocrystals were precipitated by centrifugation with the addition of ethyl acetate (40 mL), followed by dispersing in ethanol (8 mL). A small amount of 2-ethanolamine (160 μL) was then introduced into

the ZnO nanocrystals solution as surface ligands. Purified ZnO was obtained by adding ethyl acetate (40 mL) again. Next, the ZnO solution was obtained by re-dispersing in ethanol (6.4 mL). The solutions were filtered through a 0.22 μm PTFE filter before use.

References

1. Cao, Y. *et al.* Perovskite light-emitting diodes based on spontaneously formed submicrometre-scale structures. *Nature* **562**, 249–253 (2018).

2. Wang, N. *et al.* Perovskite light-emitting diodes based on solution-processed self-organized multiple quantum wells. *Nat. Photonics* **10**, 699–704 (2016).

3. Dai, X. *et al.* Solution-processed, high-performance light-emitting diodes based on quantum dots. *Nature* **515**, 96–99 (2014).

4. Zhao, B. *et al.* High-efficiency perovskite–polymer bulk heterostructure light-emitting diodes. *Nat. Photonics* **12**, 783–789 (2018).

5. Zhao, B. *et al.* Efficient light-emitting diodes from mixed-dimensional perovskites on a fluoride interface. *Nat. Electron.* **3**, 704–710 (2020).

6. Stranks, S. D. *et al.* Recombination kinetics in organic-inorganic perovskites: excitons, free charge, and subgap states. *Phys. Rev. Appl.* **2**, 034007 (2014).

7. Shao, W. & Guo, G. Multiple-try simulated annealing algorithm for global optimization. *Math. Probl. Eng.* **2018**, e9248318 (2018).

8. Sun, Y. *et al.* Bright and stable perovskite light-emitting diodes in the near-infrared range. *Nature* **615**, 830–835 (2023).

9. Ruf, F. *et al.* Temperature-dependent studies of exciton binding energy and phase-transition suppression in (Cs,FA,MA)Pb(I,Br)$_3$ perovskites. *APL Mater.* **7**, 031113 (2019).

10. Chen, T. *et al.* Origin of long lifetime of band-edge charge carriers in organic–inorganic lead iodide perovskites. *Proc. Natl. Acad. Sci. USA* **114**, 7519–7524

(2017).

11. Stoumpos, C. C., Malliakas, C. D. & Kanatzidis, M. G. Semiconducting tin and lead iodide perovskites with organic cations: phase transitions, high mobilities, and near-infrared photoluminescent properties. *Inorg. Chem.* **52**, 9019–9038 (2013).

12. Marronnier, A. *et al.* Anharmonicity and disorder in the black phases of cesium lead iodide used for stable inorganic perovskite solar cells. *ACS Nano* **12**, 3477–3486 (2018).

13. Blöchl, P. E. Projector augmented-wave method. *Phys. Rev. B* **50**, 17953–17979 (1994).

14. Zhu, L. *et al.* Unveiling the additive-assisted oriented growth of perovskite crystallite for high performance light-emitting diodes. *Nat. Commun.* **12**, 5081 (2021).

15. Wang, J. *et al.* Interfacial control toward efficient and low-voltage perovskite light-emitting diodes. *Adv. Mater.* **27**, 2311–2316 (2015).
